# Supplementary figures and images for: RNA Polymerase III subunit Polr3a is required for craniofacial cartilage and bone development in zebrafish
Source: PLoS Genet. 2026 Jun 8;22(6):e1012164. doi: 10.1371/journal.pgen.1012164 (PMC13262943; doi:10.1371/journal.pgen.1012164)

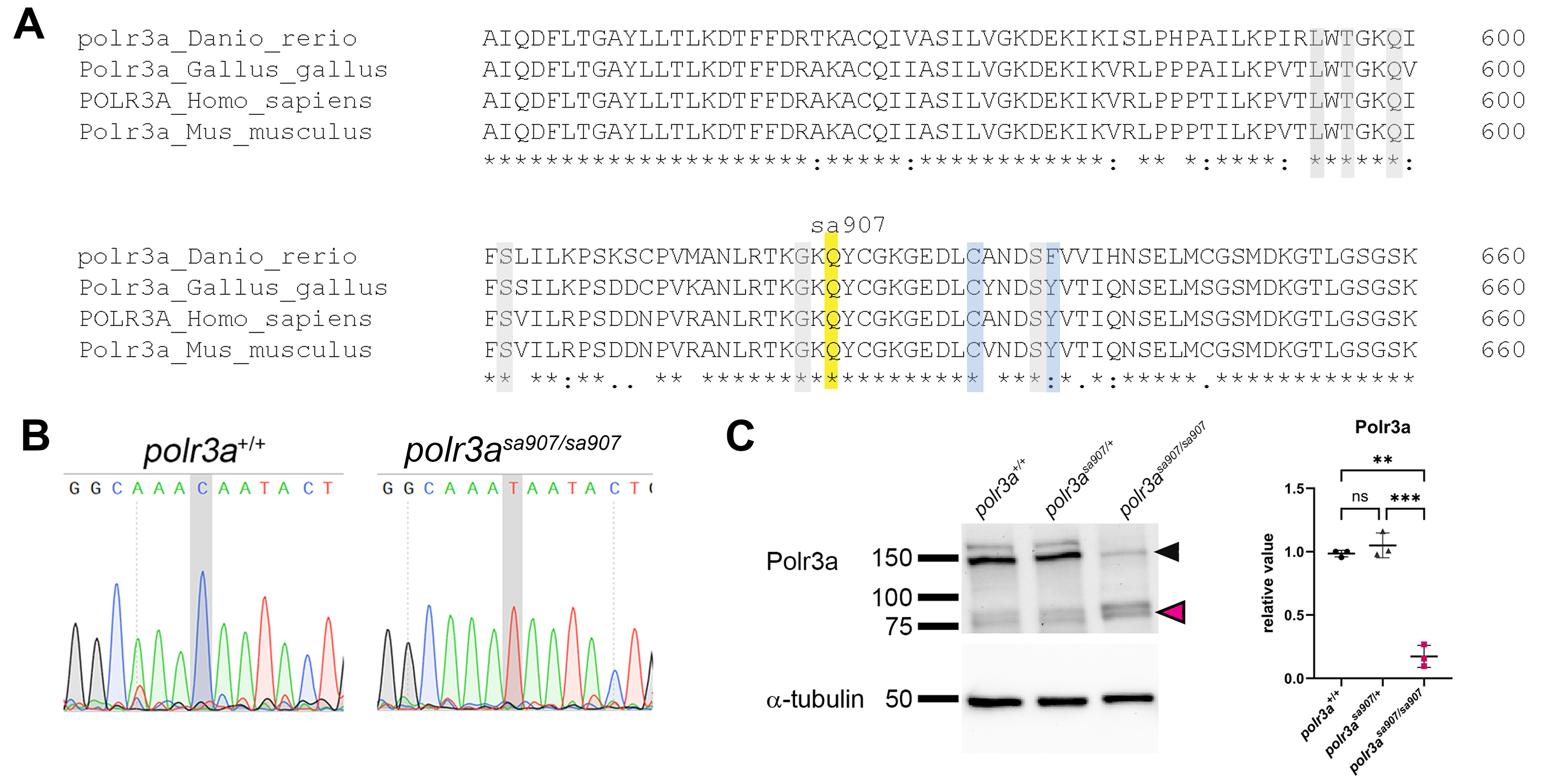

Supplement: S1 Fig — (A) The POLR3A/Polr3a protein sequence is conserved across humans, zebrafish, and other vertebrates at the site of the sa907 mutation (yellow). Locations of variants associated with POLR3-HLD (grey) and WRS (light blue) are also indicated. Amino acids 541–660 are represented here. Full length POLR3A has 1390 amino acids. (B) Sanger sequencing shows the sa907 allele is a C > T nonsense mutation in exon 14. (C) Western blotting shows a reduction in full length Polr3a in polr3asa907/sa907 zebrafish at 5 dpf (155 kDa, black arrowhead). Mutants may produce a truncated protein (78 kDa, magenta arrowhead). α-tubulin was used as a loading control. Quantification shows around a 90% reduction in Polr3a protein in polr3asa907/sa907 zebrafish. **p = 0.0087; ***p= 0.0008. (TIF) [file pgen.1012164.s001.tif]

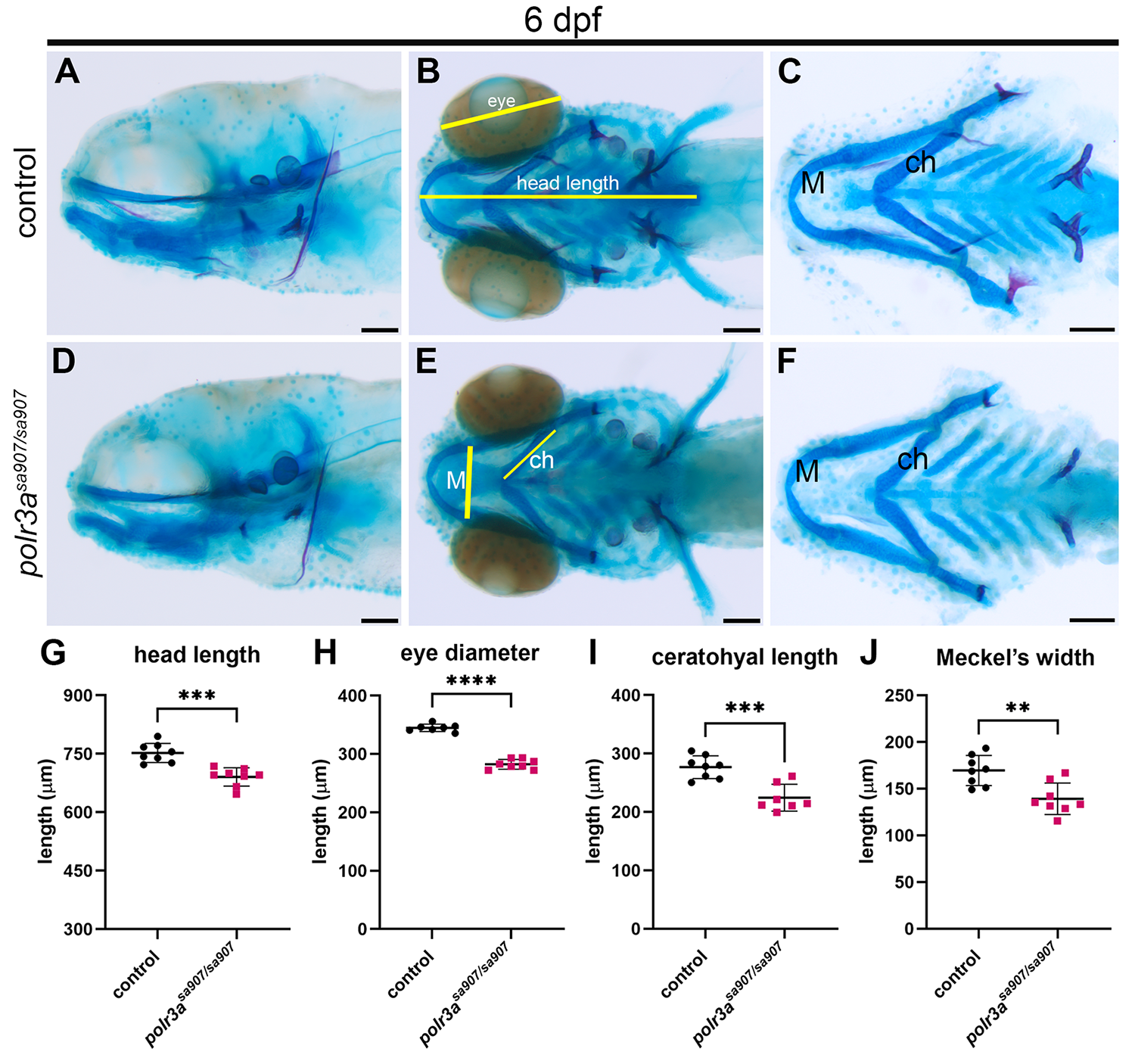

Supplement: S2 Fig — (A-F) Alizarin red and Alcian blue staining at 6 dpf reveals differences in the size of skeletal elements in mutant zebrafish. Ventral views (B,E) show overall head length (G, ***p = 0.0002) and eye diameter (H, ****p < 0.0001) are smaller (measurements indicated in panel B). The length of the ceratohyal (ch) is shorter in mutants (I, *** p = 0.0005), as is the width of Meckel’s (J, **p = 0.003) from the left to right jaw joint (measurements indicated in panel E). Abbreviations: M, Meckel’s cartilage; ch, ceratohyal. Scale bar = 100 µm. n = 8 controls, n = 8 mutants. (TIF) [file pgen.1012164.s002.tif]

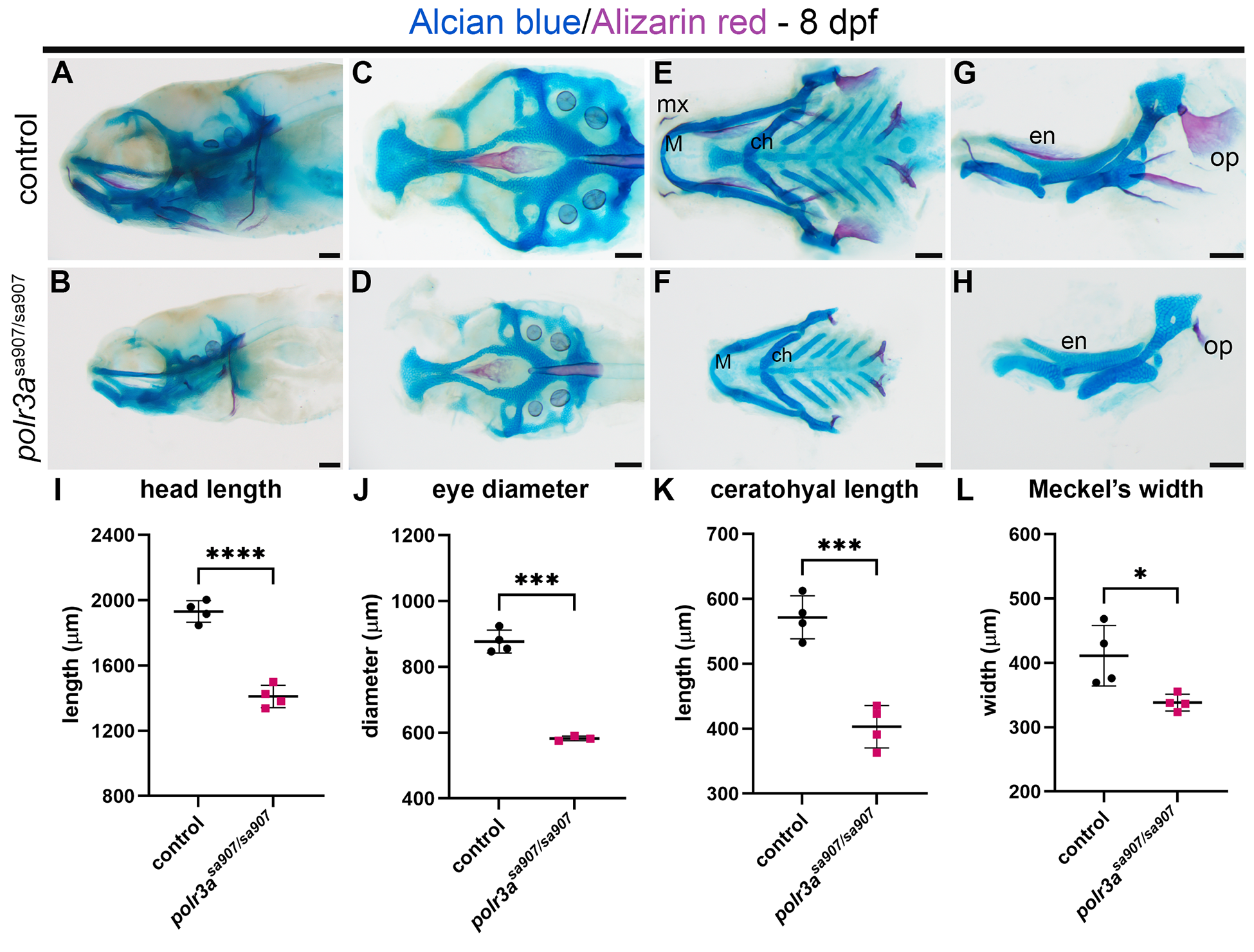

Supplement: S3 Fig — (A-H) Alizarin red and Alcian blue staining at 8 dpf demonstrates the overall reduction of skeletal elements in mutant zebrafish. (A,B) Lateral views show the size difference between control and mutant larvae. (C,D) Flat mounts of the neurocranium show reductions in cartilage formation, especially around the eye. (E,F) Flat mounts of the viscerocranium reveal the reduction of NCC-derived cartilage and bone structures including the maxilla (mx), ceratohyal (ch), and Meckel’s cartilage (M). (G,H) Dissection of arch 1 and 2-derived elements show the differences in bone at 8 dpf. The opercle (op) remains reduced in size, there is a small amount of bone forming in the entopterygoid (en) in mutants, while other bones fail to form. Overall head length (I, ***p < 0.0001) and eye diameter (J, ***p = 0.0003) are smaller. The length of the ceratohyal (ch) is shorter in mutants (I, *** p = 0.0004), as is the width of Meckel’s (J, *p = 0.048) from the left to right jaw joint (as in S2 Fig). Scale bar = 200 µm. n = 4 controls, n = 4 mutants. (TIF) [file pgen.1012164.s003.tif]

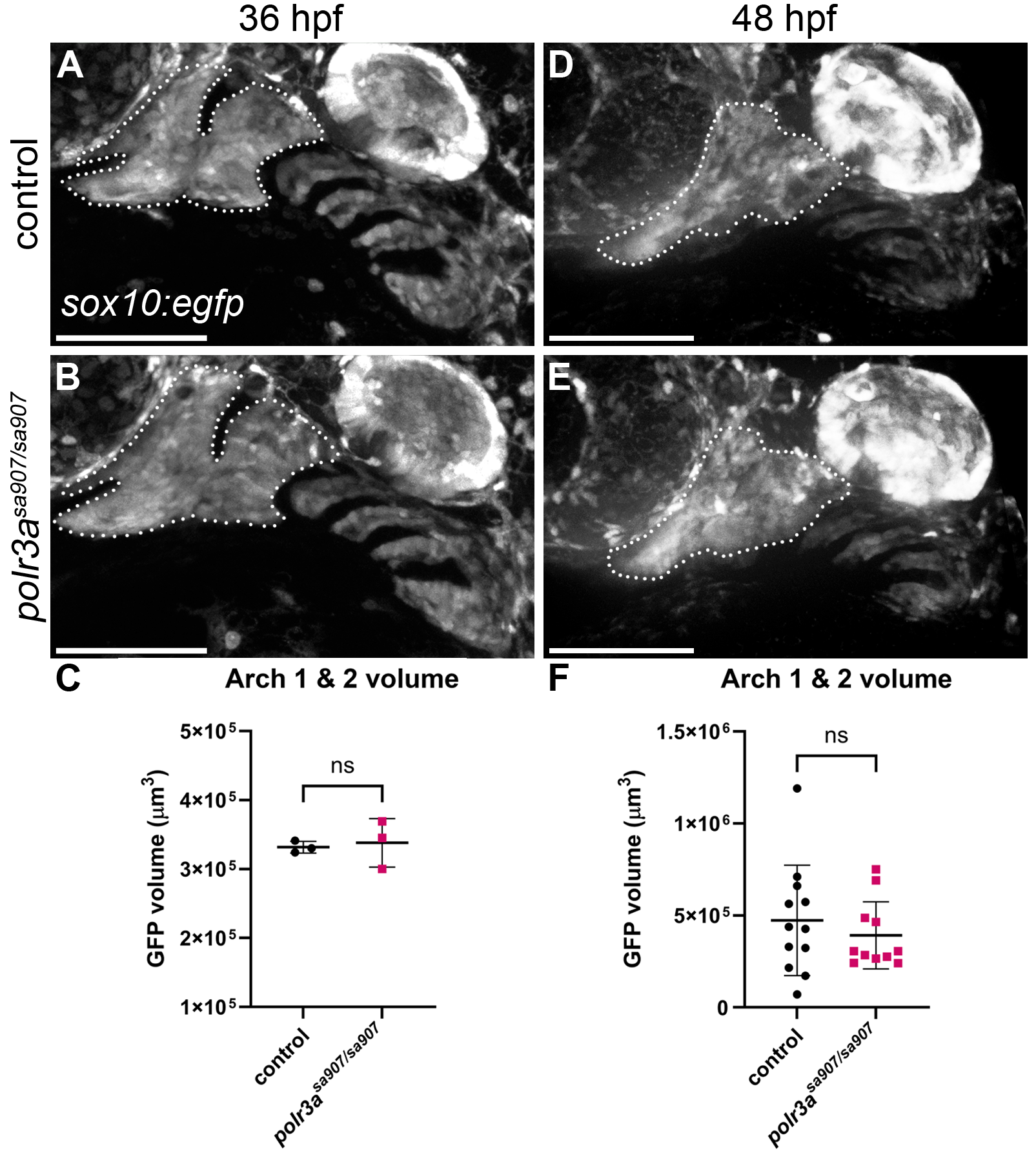

Supplement: S4 Fig — (A,B) sox10:egfp expression in control and polr3asa907/sa907 zebrafish at 36 hpf. Pharyngeal arch 1 and 2 are outlined and quantification of this region shows no significant difference between controls and mutants (C). n = 3 controls, n = 3 mutants. (D,E) sox10:egfp expression at 48 hpf also shows no significant changes in arch volume, outlined area (F). n = 12 controls, n = 11 mutants. Scale bar = 100 µm. (TIF) [file pgen.1012164.s004.tif]

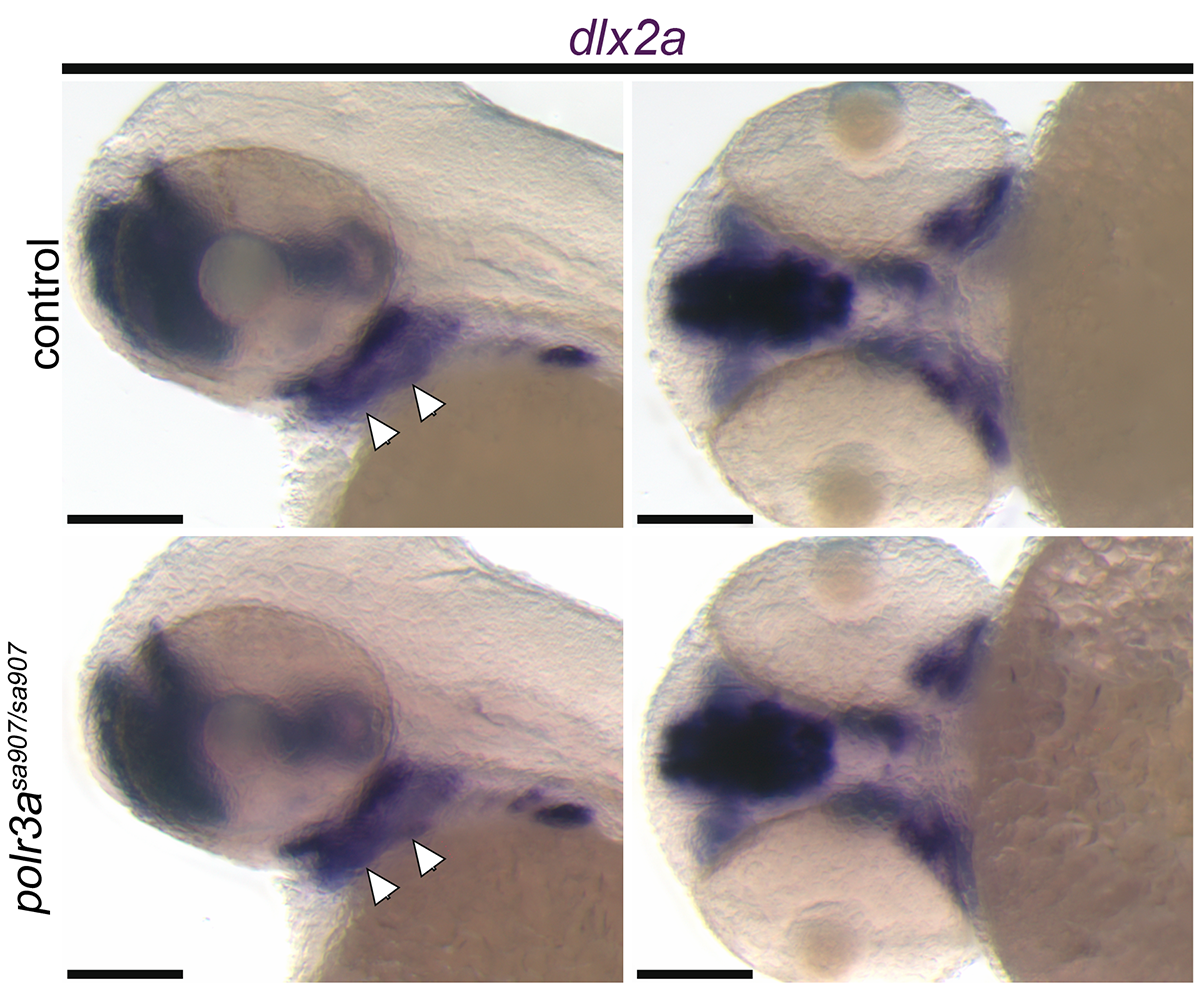

Supplement: S5 Fig — At 48 hpf, no differences were detected in dlx2a expression, as a marker of the pharyngeal arches. White arrowheads indicate anatomical location of arch 1 and 2. Scale bar = 100 µm. (TIF) [file pgen.1012164.s005.tif]

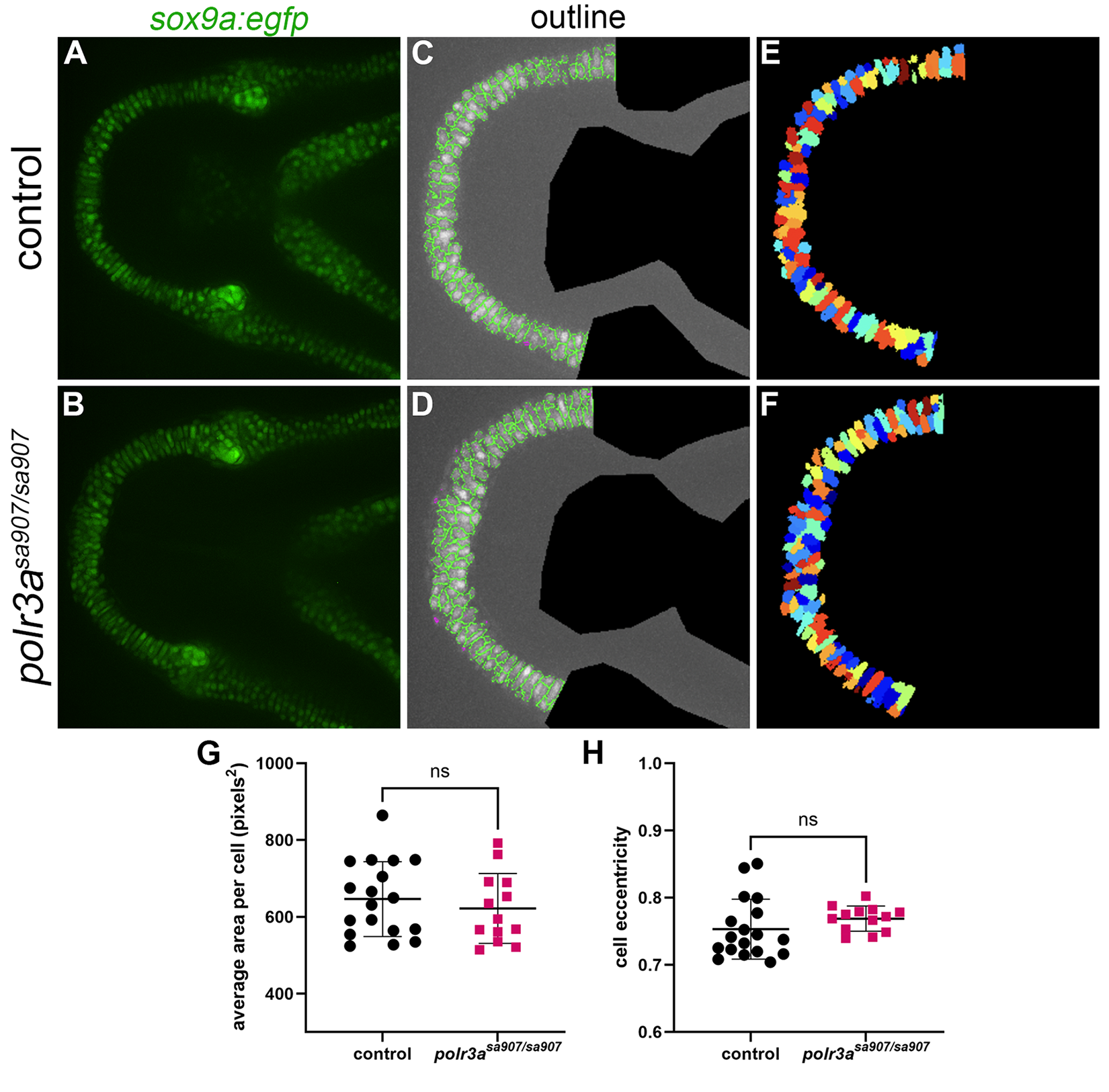

Supplement: S6 Fig — (A,B) Images of Meckel’s cartilage using sox9a:egfp expression. (C,D) Cells in Meckel’s cartilage were outlined using CellProfiler. (E,F) Representation of cell masking from CellProfiler showing the organization of cells. These masks were used to quantify cell number, area, and eccentricity. There were no differences in the average area per cell (G; p = 0.478) or in cell eccentricity (H; p = 0.196) in polr3asa907/sa907 mutants compared to controls. n = 18 controls, n = 13 mutants. (TIF) [file pgen.1012164.s006.tif]

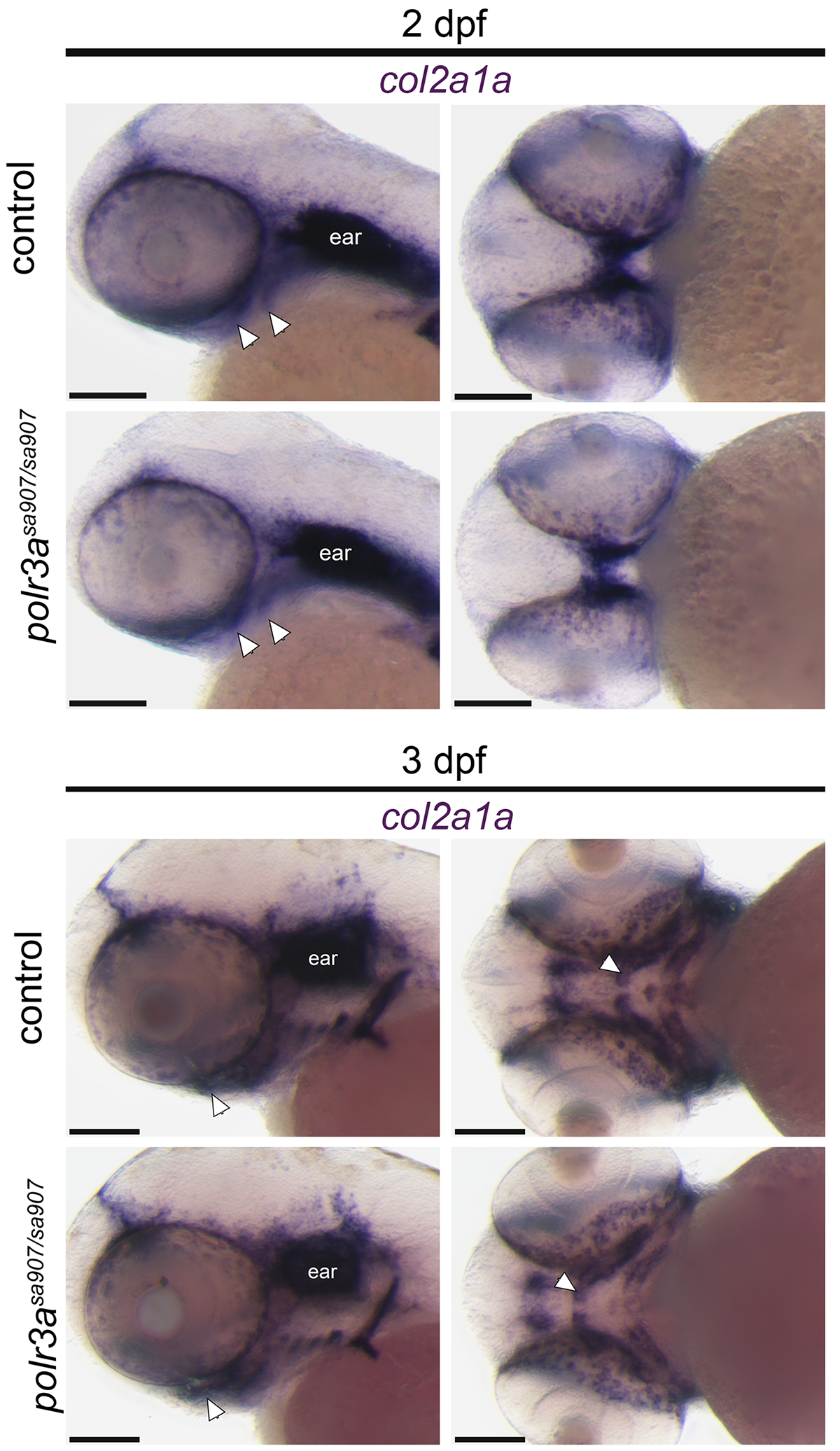

Supplement: S7 Fig — Staining in the region of arch 1 and 2-derived cartilage is unaffected (arrows). Scale bar = 100 µm. (TIF) [file pgen.1012164.s007.tif]

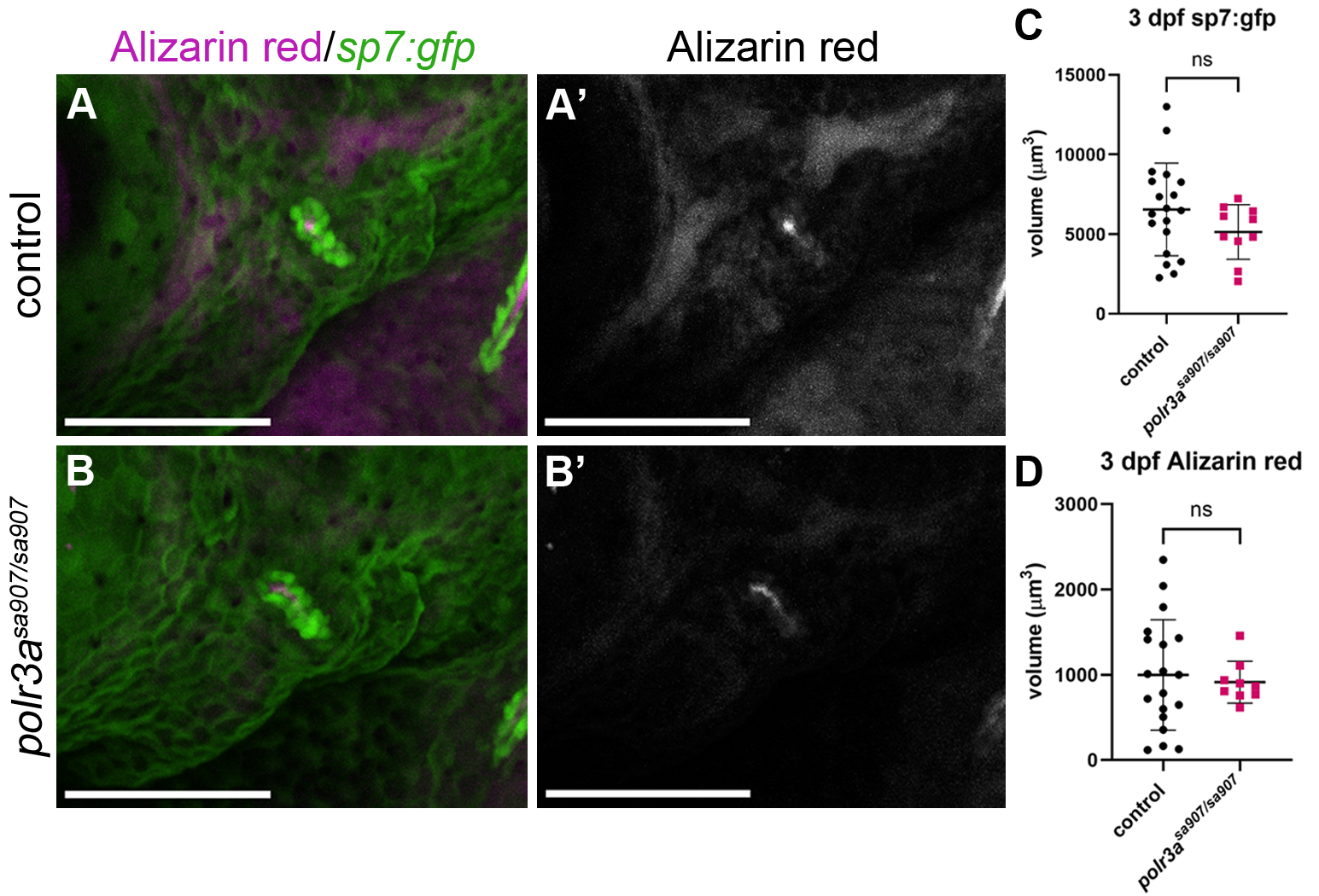

Supplement: S8 Fig — (A,B) sp7:gfp to label osteoblasts (green) and Alizarin red for bone (magenta) shows no significant changes at 3 dpf in either sp7:gfp (C) or Alizarin red (D). n = 19 controls, n = 10 mutants. Scale bar = 100 µm. (TIF) [file pgen.1012164.s008.tif]

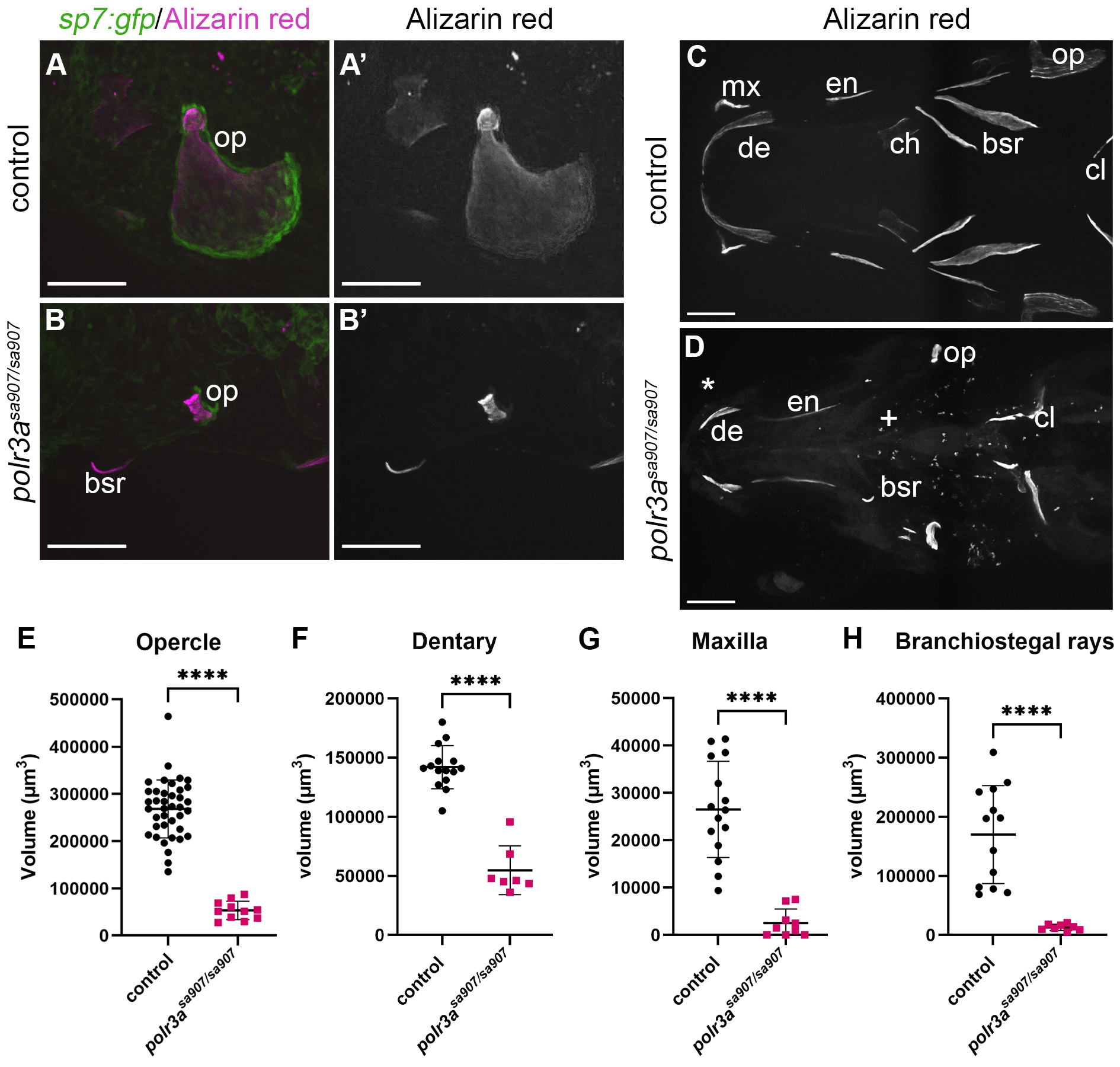

Supplement: S9 Fig — A, B) sp7:gfp to label osteoblasts (green) and Alizarin red for bone (magenta) shows a significant reduction in Alizarin red volume in the opercle (op) in mutant larvae (C; ****p < 0.0001). n = 39 controls, n = 11 polr3asa907/sa907. (D,E) Ventral views demonstrate persistence of bone hypoplasia in mutant zebrafish in all craniofacial bones examined. Quantification of the dentary (de; F; n = 15 controls, n = 7 mutants), maxilla (mx, G; n = 15 controls, n = 9 mutants), and branchiostegal rays (bsr, H; n = 13 controls, n = 8 mutants) further demonstrate that these elements remain significantly reduced. ****p < 0.0001. The maxilla was not detected in 3 larvae examined (noted by the * in D). The entopterygoid (en) remains smaller in mutant larvae. By 8 dpf, we detect little endochondral ossification in the ceratohyal (ch) in mutants, noted by the + in panel E. Scale bar = 100 µm. (TIF) [file pgen.1012164.s009.tif]

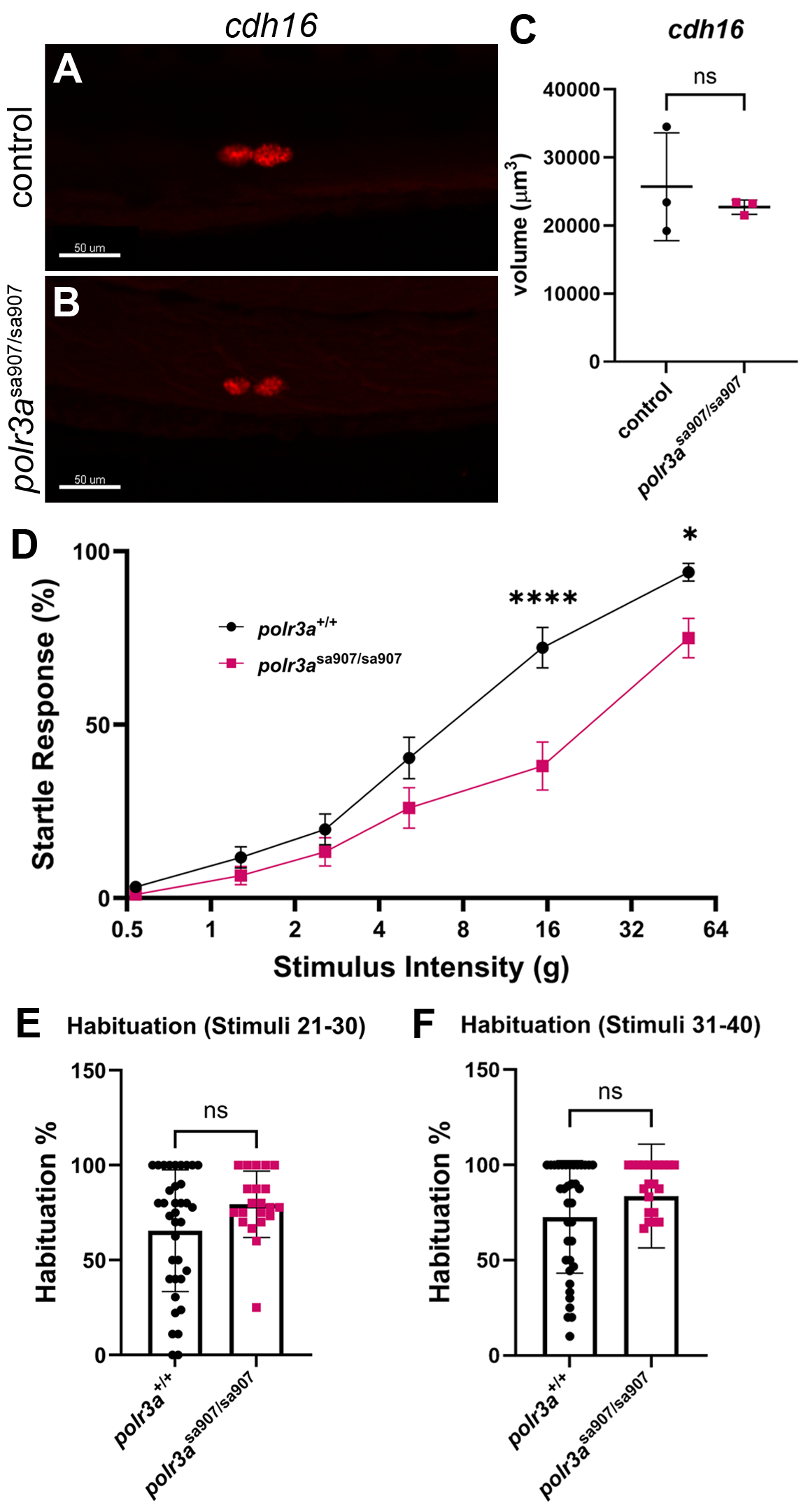

Supplement: S10 Fig — (A-C) Expression of corpuscle marker cdh16 is unchanged in polr3a mutants relative to controls. ns, not significant. (D-F) polr3asa907/sa907 mutant zebrafish are hyposensitive to acoustic stimuli. ****p < 0.0001; *p = 0.024. n = 37 controls, n = 25 mutants. Scale bar = 50 µm. (TIF) [file pgen.1012164.s010.tif]

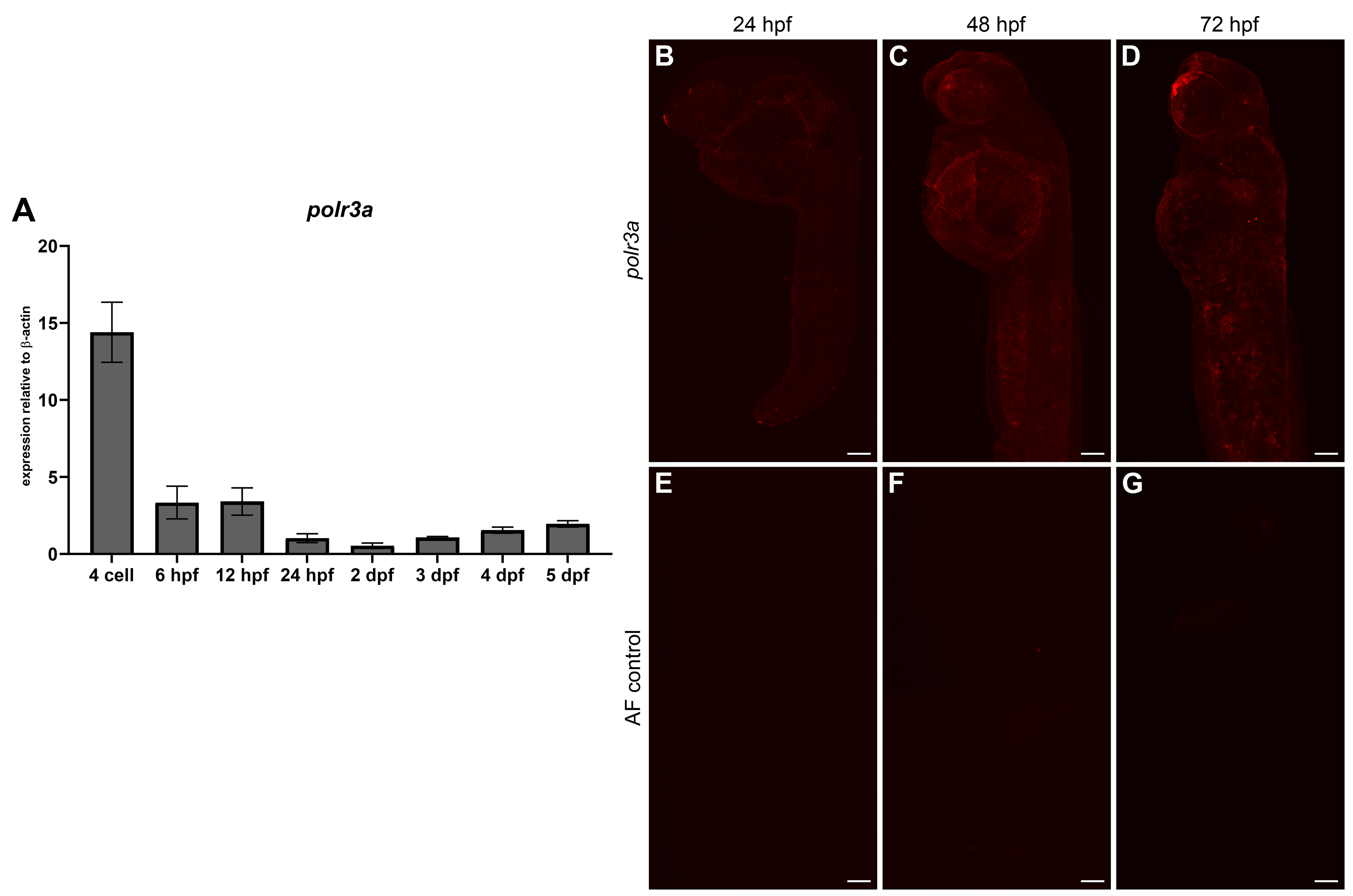

Supplement: S11 Fig — (A) qRT-PCR expression of polr3a in wild type embryos shows extensive maternal expression at 4-cell stage, and then increasing expression from 3–5 dpf. (B-D) HCR in situ for polr3a shows a broad low-level of expression at 24 hpf (B), 48 hpf (C), and 72 hpf (D), in wild-type embryos. The autofluorescence negative controls do not show expression at any stage (E-G). Scale bar = 100 µm. (TIF) [file pgen.1012164.s011.tif]

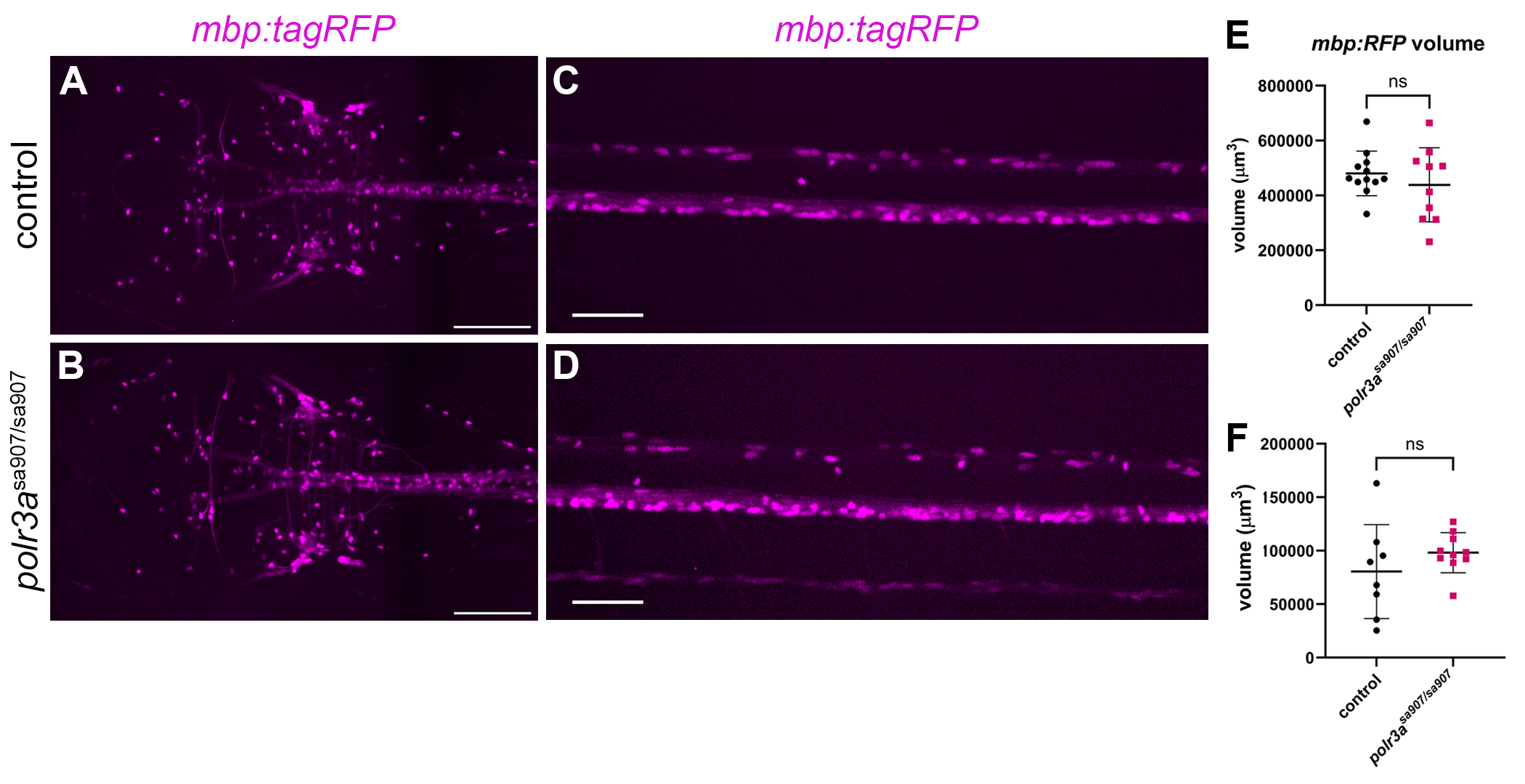

Supplement: S12 Fig — (A,B) mbp:tagRFP expression (magenta) is unchanged in the hindbrain (n = 12 controls; n = 12 mutants). (C,D) No changes in mbp:tagRFP expression in spinal cord were observed (n = 8 controls; n = 10 mutants) in polr3asa907/sa907 zebrafish. Quantification of mbp:tagRFP volume in the head (E) and spinal cord (F) shows no statistically significant differences. ns, not significant. Scale bar = 100 µm. (TIF) [file pgen.1012164.s012.tif]

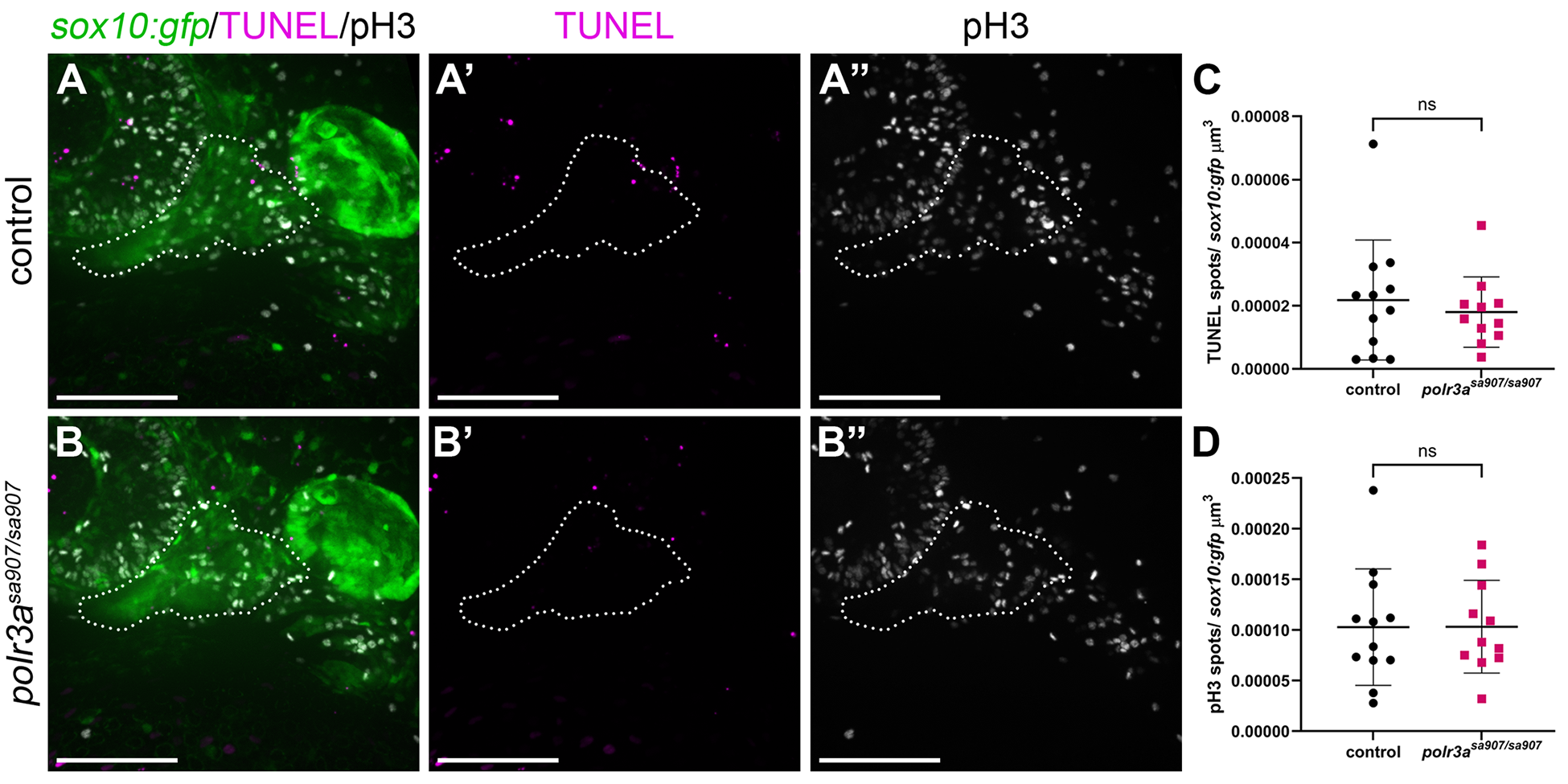

Supplement: S13 Fig — (A,B) TUNEL (magenta) and pH3 (white) within the region of arch 1 and 2 (white outline) show no significant differences between control and polr3asa907/sa907 embryos. (C) Quantification of TUNEL spots within arches 1 and 2 shows no significant difference, nor does pH3 (D). ns, not significant. n = 12 controls, n = 11 mutants. Scale bar = 100 µm. (TIF) [file pgen.1012164.s013.tif]

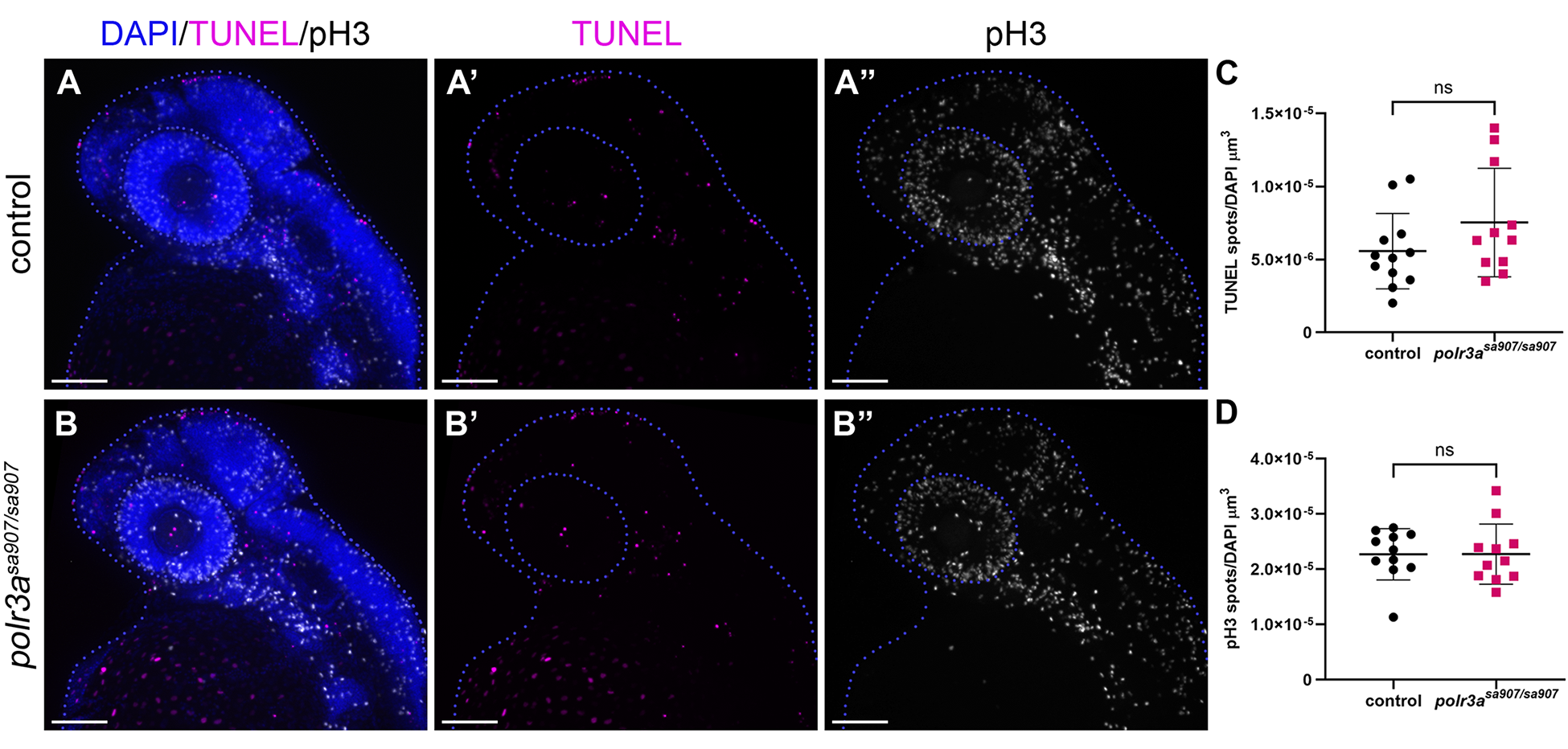

Supplement: S14 Fig — (A,B) Analysis of TUNEL (magenta) and pH3 (white) throughout the head (DAPI, all nuclei; blue outline) at 2 dpf. (C) Quantification of TUNEL and pH3 (D) reveals no significant differences between control and polr3asa907/sa907 embryos. n = 12 controls, n = 11 mutants. Scale bar = 100 µm. (TIF) [file pgen.1012164.s014.tif]

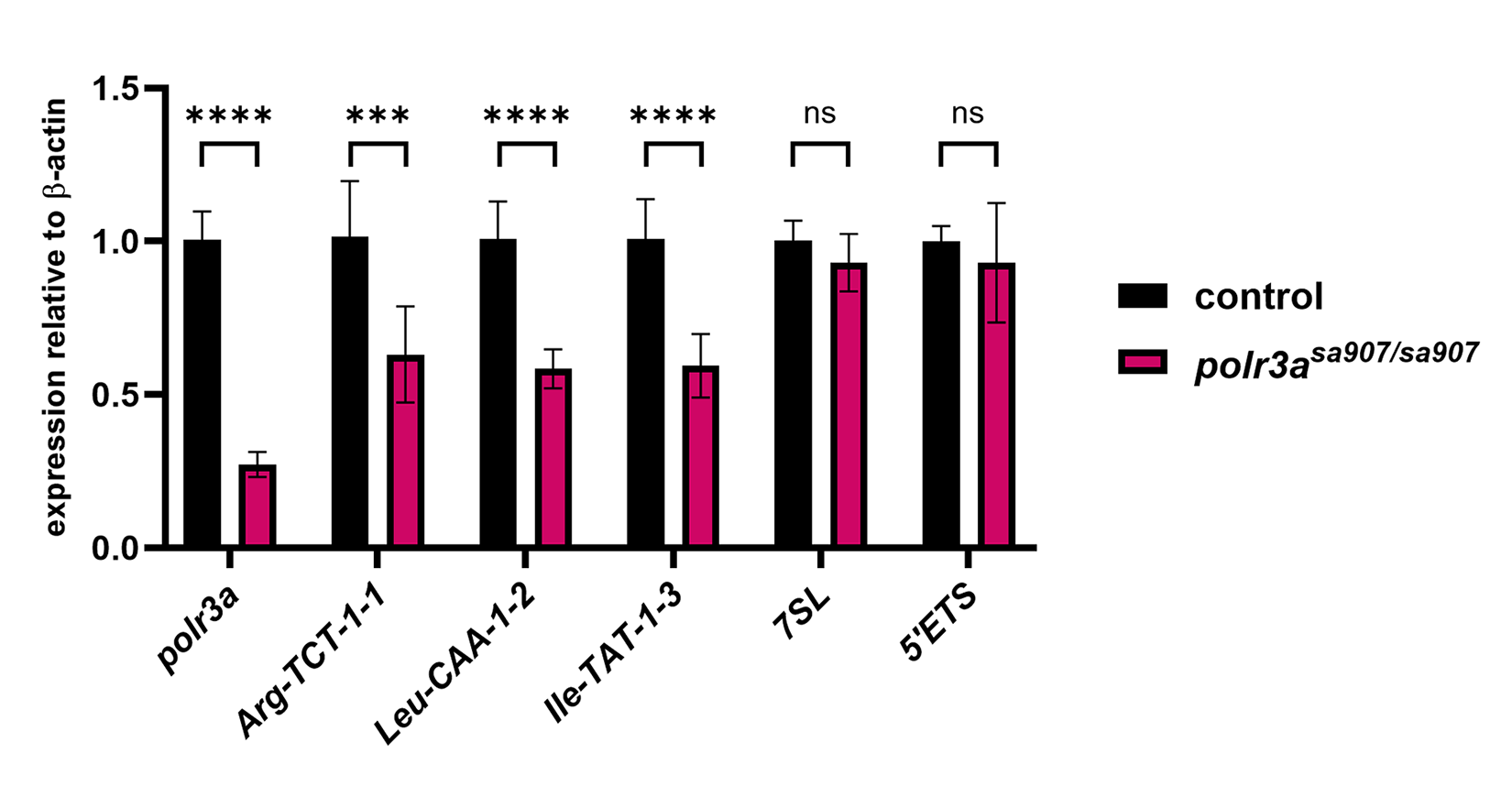

Supplement: S15 Fig — qRT-PCRs for pre-tRNAs, 7SL RNA, and 5’ETS rRNA shows that only pre-tRNAs are significantly reduced at 2 dpf. ****p < 0.0001; ***p = 0.0002. (TIF) [file pgen.1012164.s015.tif]

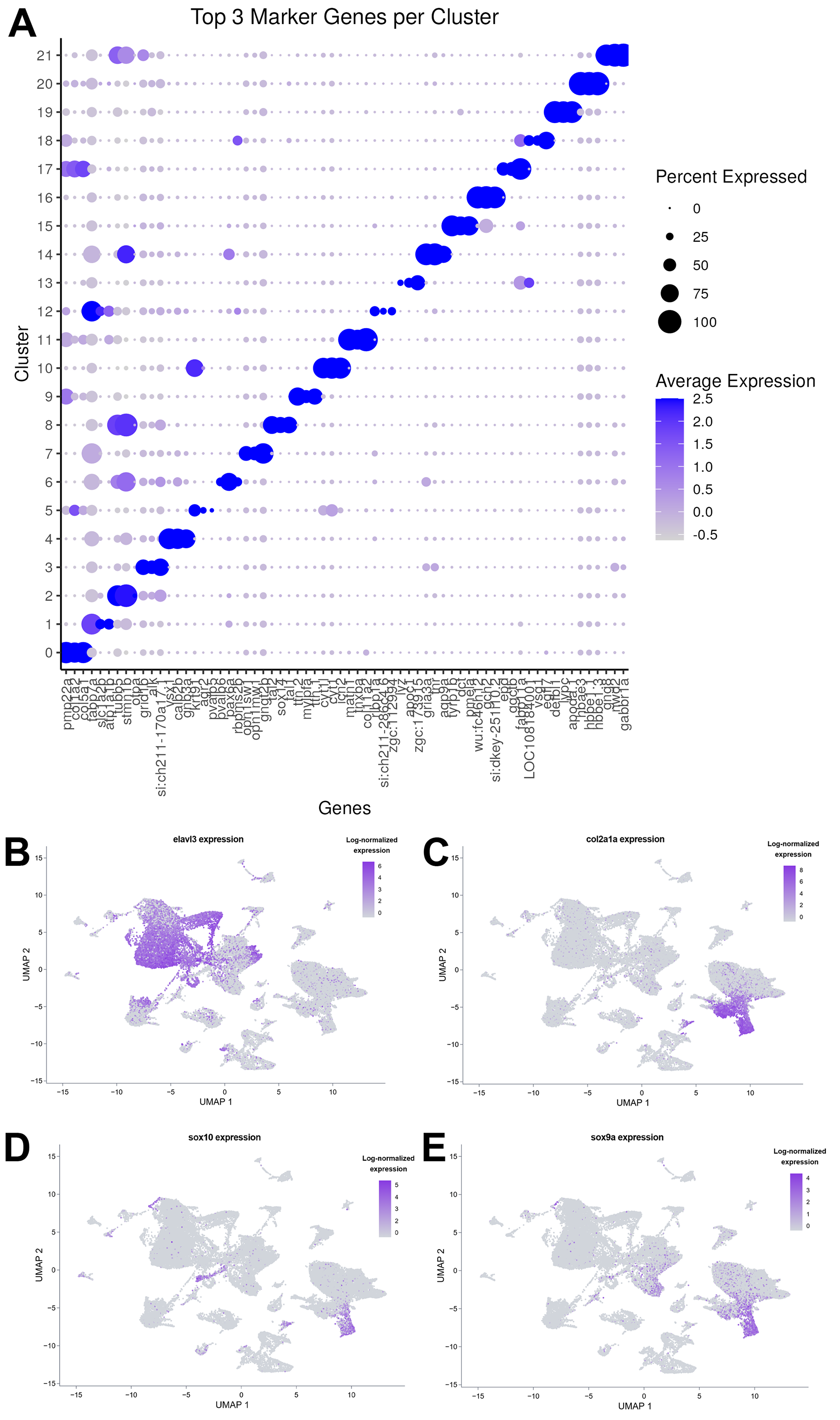

Supplement: S16 Fig — (A) Dot plot of top three marker genes for each cluster. (B) UMAP plot of elavl3 expression as a marker of neuronal cells. (C) UMAP plot of col2a1a expression as a marker of cartilage cells. (D) UMAP plot of sox10 expression. (E) UMAP plot of sox9a expression. (TIF) [file pgen.1012164.s016.tif]

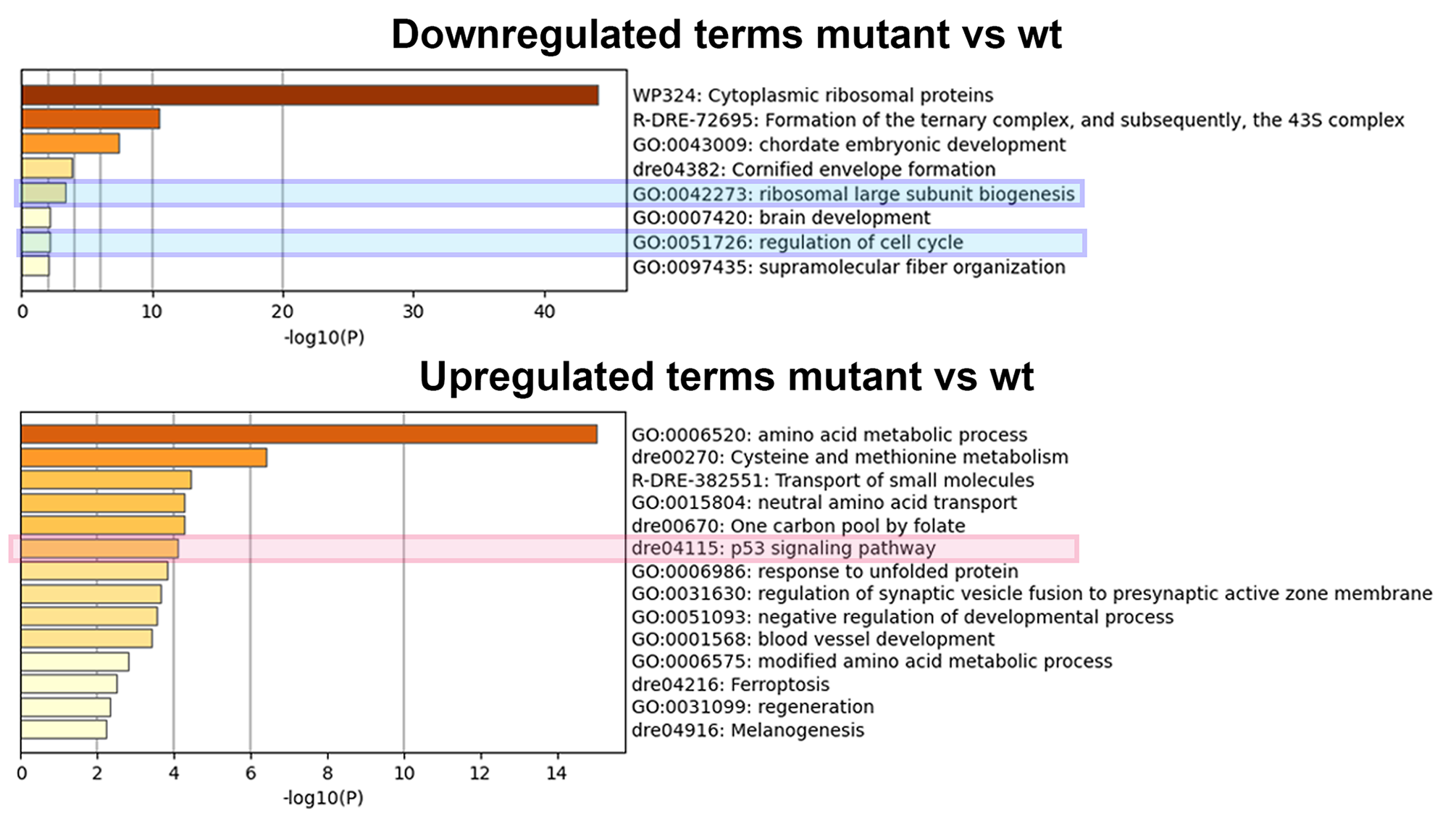

Supplement: S17 Fig — Downregulated terms include ribosome large subunit biogenesis and cell cycle (blue boxes). Terms upregulated in mutant zebrafish include Tp53 signaling (magenta box). (TIF) [file pgen.1012164.s017.tif]

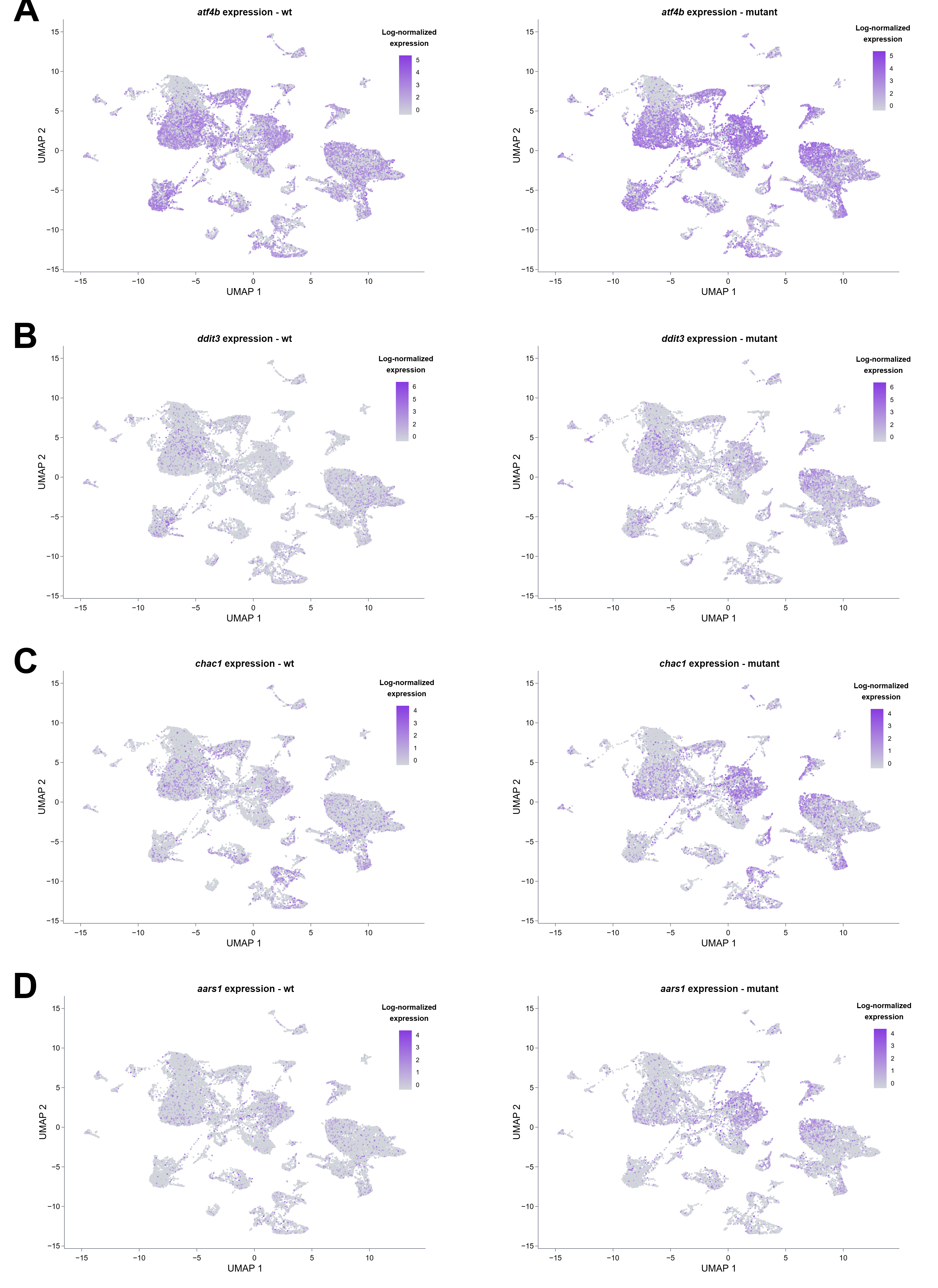

Supplement: S18 Fig — The polr3a+/+ sample (wt) is on the left and the polr3asa907/sa907 sample (mutant) is on the right. (A) Expression of atf4b. (B) Expression of ddit3. (C) Expression of chac1. (D) Expression of aars1. (TIF) [file pgen.1012164.s018.tif]

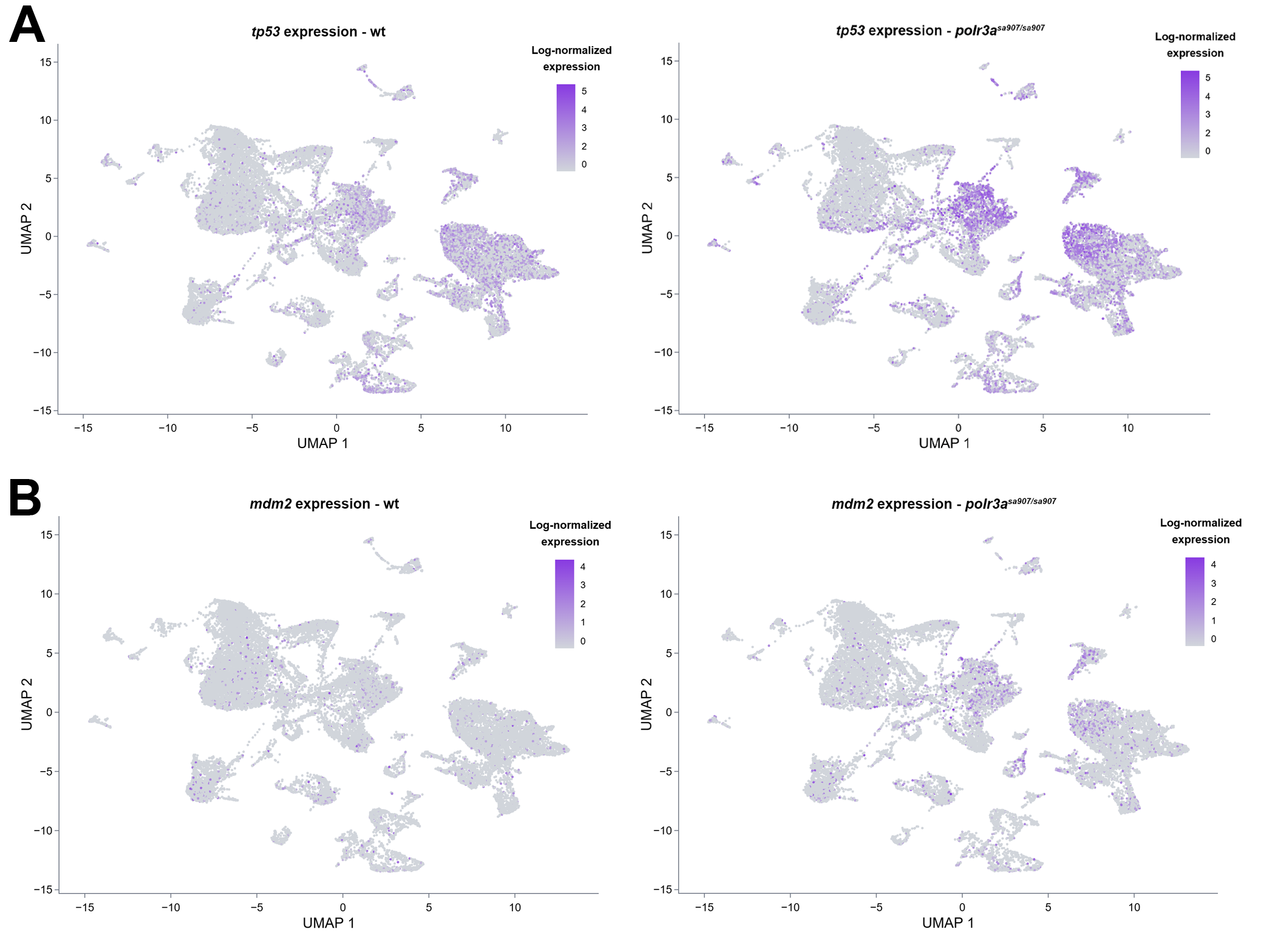

Supplement: S19 Fig — (A) UMAP plots of tp53 expression and (B) mdm2 expression in wild-type (wt, left) versus polr3asa907/sa907 zebrafish (right). (TIF) [file pgen.1012164.s019.tif]

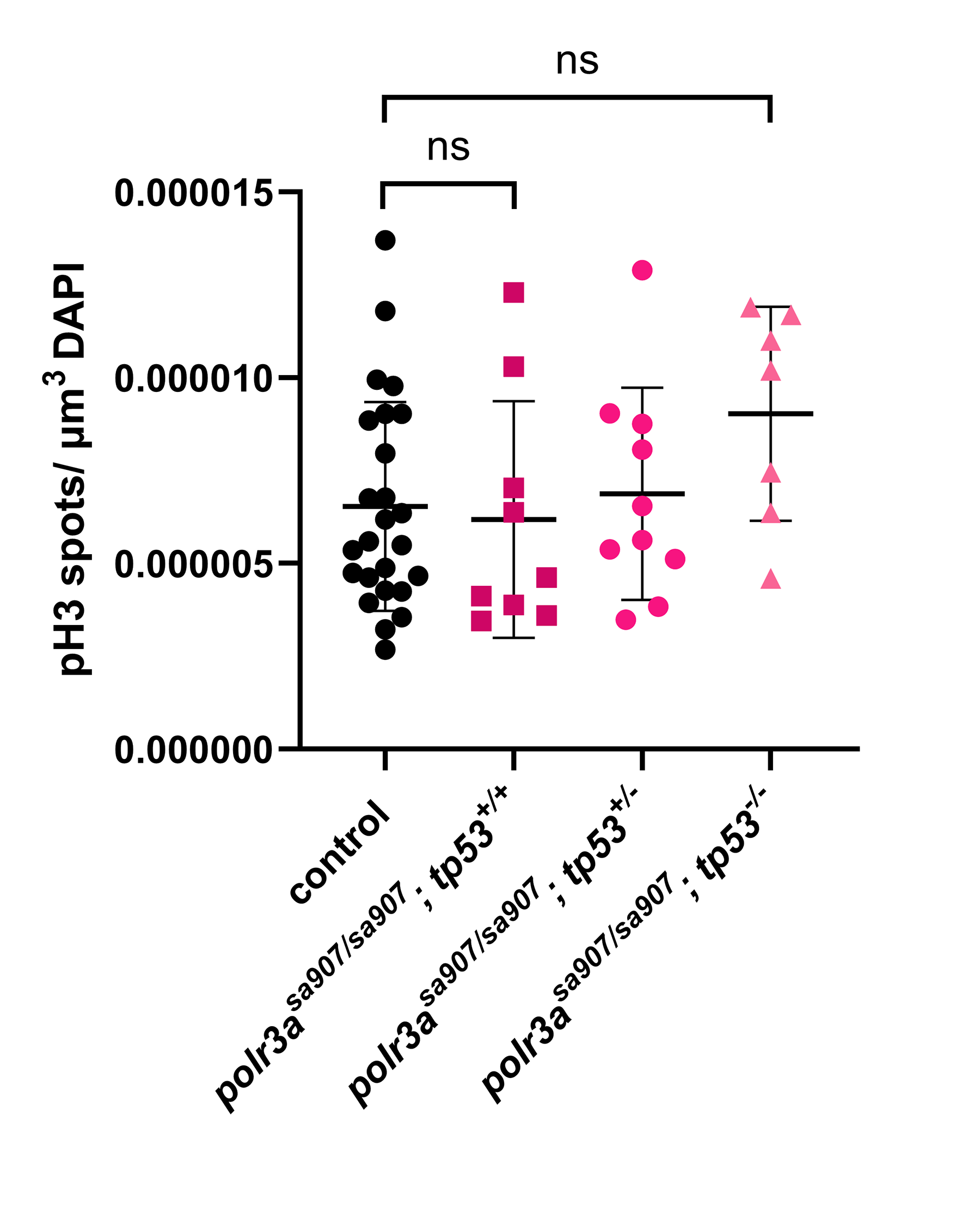

Supplement: S20 Fig — (TIF) [file pgen.1012164.s020.tif]

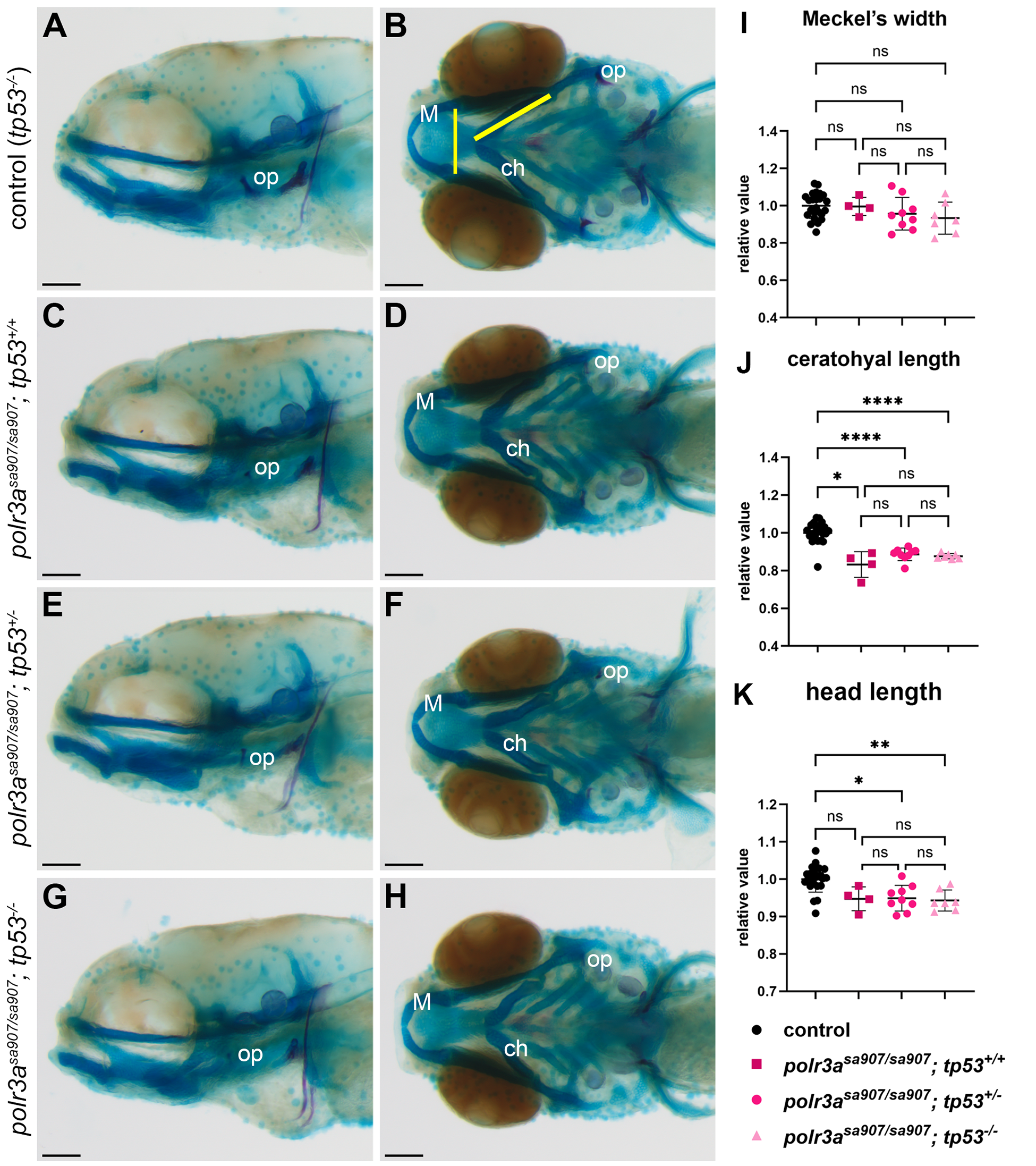

Supplement: S21 Fig — (A,B) Control zebrafish show bone staining in the opercle and organized stacking in Meckel’s cartilage. polr3asa907/sa907; tp53+/+ zebrafish have smaller opercles (C,D) as do polr3asa907/sa907; tp53+/- (E,F) and polr3asa907/sa907; tp53-/- (G,H) zebrafish. Quantification of the width of Meckel’s cartilage (vertical yellow line in B) reveals no significant changes (I). Quantification of ceratohyal length (J), demonstrates no significant improvements in length regardless of tp53 status. Adjusted p-values: ns, not significant. *p = 0.04; ****p < 0.0001. (K) Head length remains reduced in polr3asa907/sa907 zebrafish with inhibition of tp53. Adjusted p-values: ns, not significant; *p = 0.01; **p = 0.004. n = 24 control, n = 4 pol3asa907/sa907; tp53+/+; n = 9 polr3asa907/sa907; tp53+/-; n = 7 polr3asa907/sa907; tp53-/-. ch, ceratohyal; M, Meckel’s cartilage; op, opercle. Scale bar = 100 µm. (TIF) [file pgen.1012164.s021.tif]

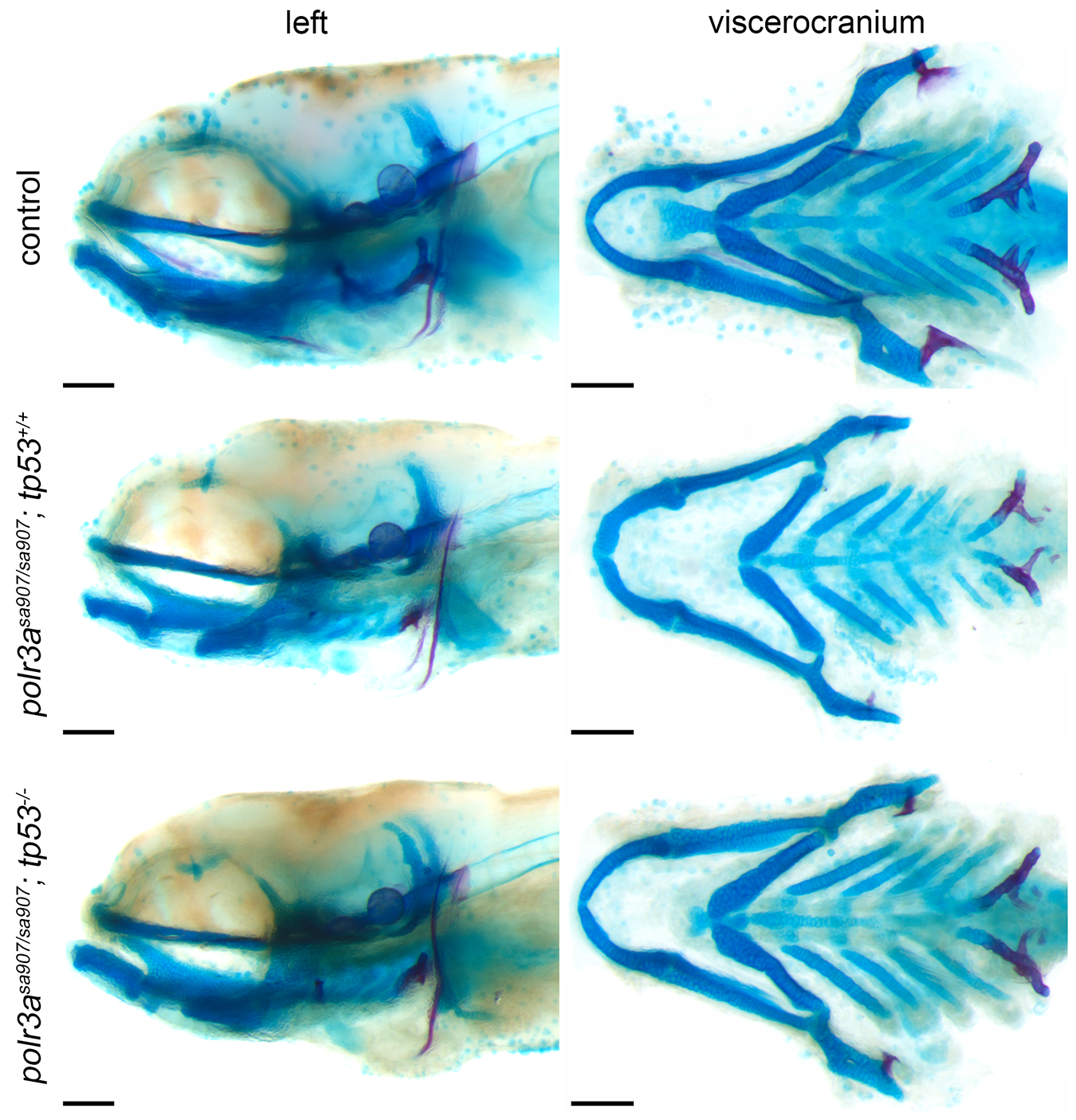

Supplement: S22 Fig — (TIF) [file pgen.1012164.s022.tif]
